# The quantitative impact of COVID-19 on surgical training in the United Kingdom

**DOI:** 10.1093/bjsopen/zrab051

**Published:** 2021-06-25

**Authors:** J M Clements, J R Burke, C Hope, D M Nally, B Doleman, L Giwa, G Griffiths, J N Lund

**Affiliations:** The Association of Surgeons in Training, London, UK; The Association of Surgeons in Training, London, UK; Division of Medical Sciences and Graduate Entry Medicine, School of Medicine, Royal Derby Hospital, Derby, UK; The Association of Surgeons in Training, London, UK; Division of Medical Sciences and Graduate Entry Medicine, School of Medicine, Royal Derby Hospital, Derby, UK; The Association of Surgeons in Training, London, UK; Joint Committee on Surgical Training, London, UK; Division of Medical Sciences and Graduate Entry Medicine, School of Medicine, Royal Derby Hospital, Derby, UK; Joint Committee on Surgical Training, London, UK

## Abstract

**Background:**

COVID-19 has had a global impact on all aspects of healthcare including surgical training. This study aimed to quantify the impact of COVID-19 on operative case numbers recorded by surgeons in training, and annual review of competency progression (ARCP) outcomes in the UK.

**Methods:**

Anonymized operative logbook numbers were collated from electronic logbook and ARCP outcome data from the Intercollegiate Surgical Curriculum Programme database for trainees in the 10 surgical specialty training specialties.

Operative logbook numbers and awarded ARCP outcomes were compared between predefined dates. Effect sizes are reported as incident rate ratios (IRR) with 95 per cent confidence intervals.

**Results:**

Some 5599 surgical trainees in 2019, and 5310 in surgical specialty training in 2020 were included. The IRR was reduced across all specialties as a result of the COVID-19 pandemic (0.62; 95 per cent c.i. 0.60 to 0.64). Elective surgery (0.53; 95 per cent c.i. 0.50 to 0.56) was affected more than emergency surgery (0.85; 95 per cent c.i. 0.84 to 0.87). Regional variation indicating reduced operative activity was demonstrated across all specialties. More than 1 in 8 trainees in the final year of training have had their training extended and more than a quarter of trainees entering their final year of training are behind their expected training trajectory.

**Conclusion:**

The COVID-19 pandemic has had a major effect on surgical training in the UK. Urgent, coordinated action is required to minimize the impacts from the reduction in training in 2020.

## Teaser

This study reports the impact of the COVID-19 pandemic on operative surgical training in the United Kingdom, demonstrating significant reduction in operative training, especially in elective procedures, across the UK, and a consequent impact on trainee progression, as measured by ARCP outcome.

## Introduction

The coronavirus 2019 (COVID-19) pandemic has had a profound impact on all aspects of healthcare. In the UK, the National Health Service (NHS) began restructuring and reprioritizing services in February 2020. Elective surgical procedures were cancelled from 17 March 2020 in order to free inpatient and critical care capacity^1^. The COVIDSurg Global Collaborative study estimated the UK would cancel or postpone more than 40 000 elective operations per week during the peak of the pandemic^2^.

Throughout the first wave of the pandemic there were other changes to surgical services, including redeployment of surgical staff to support the response to COVID-19 and redirection of some elective operations from NHS hospitals, which deliver virtually all recognized training in the UK, to independent sector facilities. In the absence of a national regulatory framework for training outside the NHS, these additional factors further reduced opportunities for training and career progression. The hashtag #NoTrainingTodayNoSurgeonsTomorrow gained traction on social media, highlighting the consequences of pandemic-related reduction in training on the supply of surgical trainees to the consultant workforce if the training gap were not addressed.

Much of the early qualitative data of surgical trainee experiences in other countries present common themes involving redeployment, reduction in elective and emergency operating to varying degrees, a negative impact on mental health and wellbeing and concern about potential delays to the completion of training^3–6^. These experiences are shared among high- and low–middle-income countries^7^ and have been most recently summarized in a systematic review of 29 qualitative trainee surveys of more than 5000 trainees in 20 countries^8^. This review highlighted the magnitude and scale of the problem in all parts of the globe across various surgical specialties. Additionally, some international studies have suggested that senior surgical trainees may be disproportionately affected by the pandemic with concerns over the ability to meet operative case requirements^9^ that in turn may affect future job prospects^10^.

In the UK training system, many specialties describe indicative numbers of common procedures likely to reflect adequate levels of competence and experience. Achieving indicative numbers of index operative procedures has been identified as a challenge for trainees, in the immediate years preceding the pandemic^11^. Some steps have already been taken by Surgical Royal Colleges in the British Isles, employers, regulators and agencies involved in the delivery of surgical training to address existing problems, but it was recognized early in the pandemic that urgent additional measures would be necessary regarding promotion, competency and assessment to ensure that individual trainees were not disadvantaged by loss of surgical activity^12^. While the NHS planned to increase surgical throughput to 80 per cent of normal by September 2020^13^, a second wave of coronavirus infections in the autumn of that year made this impossible.

Surgical training in the UK and Republic of Ireland is overseen by the Joint Committee of Surgical Training (JCST) on behalf of the Surgical Royal Colleges of the UK and Ireland. JCST receives advice from its Specialty Advisory Committees and works alongside the UK General Medical Council, the four UK Statutory Education Bodies (SEBs) and Representative Trainee Associations to set the requirements for, and ensure quality of, surgical training. There are 10 recognized surgical specialty training pathways. Two years spent completing the Core Surgical Training (CST) curriculum are followed by an indicative 5 or 6 years (depending on specialty) of specialty training^14^. The Intercollegiate Surgical Curriculum Programme (ISCP) is a national pan-speciality online training management platform under the auspices of the JCST (https://www.iscp.ac.uk). Enrolment with ISCP is mandatory for trainees who use it as an electronic portfolio and platform for workplace-based assessment. Operative experience is recorded in an electronic logbook (e-logbook) which links with ISCP (https://www.elogbook.org). The annual review of competence progression (ARCP), led by local offices of the SEBs, makes decisions on progression or otherwise of trainees towards completion of training after review of evidence recorded in ISCP.

Due to the disruption to training caused by COVID-19, the SEBs recognized that there may be challenges for trainees and trainers in receiving and delivering training and in preparing and providing evidence for the ARCP during the pandemic. To ensure progression through clinical training, without detriment to the trainee where possible, ARCP Outcome 10, a no-fault outcome that takes into consideration the impact of the pandemic, was introduced^15^. Outcome 10.1 can be awarded when the trainee has been making satisfactory progress to date but there has been a delay in achieving competencies due to COVID-19. Outcome 10.2 recognizes prior satisfactory performance but there has been a delay in achieving competencies due to COVID-19 and the trainee is at a critical progression point and therefore requires additional training time^16^. Standard ARCP outcomes definitions are shown in *Table S1*^17^.

The aim of this study was to quantify the impact of COVID-19 on surgical training in the UK in terms of e-logbook recorded experience and ARCP progression decisions until the end of December 2020, in order to understand the scale of the problem and the extent of measures that might be needed in future.

## Methods

A comparative study of operative caseloads before and during the pandemic was performed. Numbers of cases recorded in e-logbook, by elective and emergency case code and UK region, were obtained from 1 October 2018 to 31 December 2020. ARCP outcomes of surgical trainees were obtained for the time from 1 April 2019 to 30 September 2019 and the same period in 2020 from the ISCP database. Study approval was granted by the ISCP Data Analysis and Audit Group (DAARG), adhering to ISCP data sharing policy. All data were anonymized. The study did not require formal ethical approval. Outcome measures were numbers of logged operative procedures and number of awarded ARCP outcomes. Numbers of operative procedures were logged by region and speciality. Operative numbers reported between 1 April 2019 and 31 December 2019 were compared with the same period in 2020. The manuscript was prepared according to the ‘strengthening the reporting of observational studies in epidemiology’ (STROBE) statement^18^.

### Statistical analysis

Numbers of procedures logged by each region per month were used to see if the incidence of procedures had decreased. The analysis was performed using negative binomial regression due to overdispersion in the data. Clustered standard errors by region were used for univariable analysis and robust standard errors for multivariable analysis. Results are presented as incident rate ratios (IRR) with 95 per cent confidence intervals, where IRR is the ratio of the number of procedures over the time period of April to December in 2020 compared with 2019. For example, an IRR of 0.50 means half the number of procedures occurred over this period in 2020 compared with 2019. Time was modelled by univariable analysis then the interaction between time and region (least affected region as reference group). Regions were defined as more affected if the region had a significant interaction with time when compared with the reference region (*P* < 0.050). Subgroup analysis examined elective *versus* emergency procedures. For orthopaedics, trauma surgery was classed as the emergency procedure. Line graphs were produced to show variations in elective and emergency procedures over time. For the ARCP analyses, trainees were considered in two groups: those in core (CT1, CT2) with the first 2 years of specialty (ST1, ST2) training and then those in the subsequent years of specialty training (ST3 onwards), described as higher surgical trainees (HST). All analyses were conducted using Stata^TM^ (StataCorp LLC, College Station, TX, USA) version 16.1.

## Results

### Operative logbook

A significant reduction in recorded operative experience was observed in 2020 compared with 2019 as shown by the overall IRR of 0.62 (95 per cent c.i. 0.60 to 0.64). Of the major specialties, vascular surgery (0.71; 95 per cent c.i. 0.64 to 0.78) and neurosurgery (0.71; 95 per cent c.i. 0.68 to 0.75) were least affected, whilst otolaryngology was most affected (0.53; 95 per cent c.i. 0.50 to 0.56). Elective case numbers (0.53; 95 per cent c.i. 0.50 to 0.56) were affected more than emergencies (0.85; 95 per cent c.i. 0.84 to 0.87). The greatest elective reduction was seen in trauma and orthopaedics (0.42; 95 per cent c.i. 0.38 to 0.48) with urology (0.64; 95 per cent c.i. 0.59 to 0.68) least affected. Emergency procedures were not reduced in oral and maxillofacial surgery (1.04; 95 per cent c.i. 0.95 to 1.15), otolaryngology (0.96; 95 per cent c.i. 0.91 to 1.00), paediatrics (0.95; 95 per cent c.i. 0.86 to 1.05), vascular (0.96; 95 per cent c.i. 0.89 to 1.04) and urology (1.04; 95 per cent c.i. 0.98 to 1.11). The greatest impact in emergency procedures was observed in cardiothoracic surgery (0.77; 95 per cent c.i. 0.61 to 0.98).

*Table I* shows the overall, elective and emergency IRR for each specialty demonstrating the reduction in case volume in the study period from 2019 to 2020. Elective operative activity has not returned to baseline in any specialty and demonstrated a second dip associated with the second wave of the pandemic in the UK.

**Table 1:** Incident rate ratios for logged procedures by specialty for April to December 2019 *versus* the same period in 2020

	All specialties	Cardiothoracic surgery	General surgery	Otolaryngology	Neuro surgery	Maxillofacial surgery	Paediatric surgery	Plastic surgery	Trauma and orthopaedics	Urology	Vascular surgery
**Overall IRR**	0.62 (0.60, 0.64)	0.64 (0.59, 0.70)	0.64 (0.61, 0.67)	0.53 (0.50, 0.56)	0.71 (0.68, 0.75)	0.56 (0.52, 0.61)	0.66 (0.61, 0.71)	0.69 (0.67, 0.70)	0.60 (0.57, 0.63)	0.67 (0.63, 0.71)	0.71 (0.64, 0.78)
**IRR elective cases**	0.53 (0.50, 0.56)	0.61 (0.56, 0.67)	0.55 (0.51, 0.59)	0.49 (0.46, 0.52)	0.63 (0.59, 0.66)	0.51 (0.47, 0.56)	0.56 (0.52, 0.59)	0.59 (0.57, 0.62)	0.42 (0.38, 0.48)	0.64 (0.59, 0.68)	0.59 (0.53, 0.67)
**IRR emergency cases**	0.85 (0.84, 0.87)	0.77 (0.61, 0.98)	0.82 (0.79, 0.85)	0.96 (0.91, 1.00)	0.87 (0.81, 0.94)	1.04 (0.95, 1.15)	0.95 (0.86, 1.05)	0.89 (0.85, 0.93)	0.83 (0.81, 0.85)	1.04 (0.98, 1.11)	0.96 (0.89, 1.04)

Values in parentheses are 95 per cent confidence intervals. IRR, incident rate ratios.

### Regional impact comparisons

**Fig. 1:**
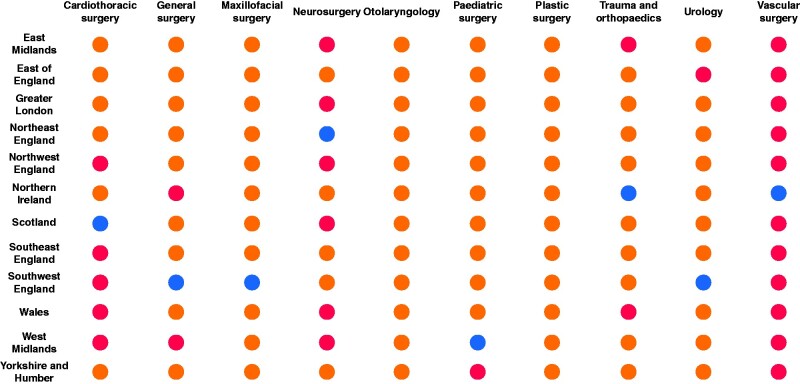
Regional variation of the impact from the COVID-19 pandemic compared with 2019 Blue indicates the region used as the reference for that speciality (least affected region). Red indicates regions experiencing significantly more impact than the reference region (*P* < 0.050). Orange colour indicates no statistically significant difference in impact between region and the reference region

*Figure 1* outlines the regional variation of the impact of the COVID-19 pandemic by speciality in 2020. Wales and the West Midlands had the most specialties with greater reductions than the reference region (4 specialties), followed by the East Midlands and Northwest England respectively (3 specialties).

### Annual review of competency progression

**Fig. 2:**
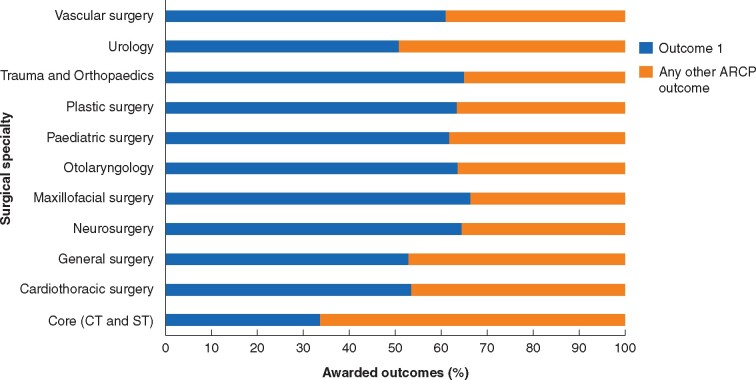
Awarded annual review of competency progression outcomes by specialty between April and September 2019 CT, core training; ST, specialty training; ARCP, annual review of competency progression

**Fig. 3:**
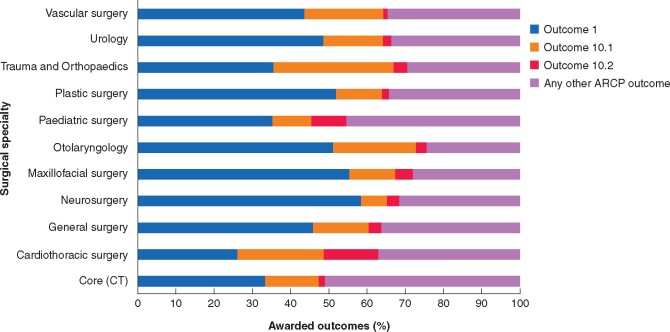
Awarded annual review of competency progression outcomes by specialty between April and September 2020 CT, core training; ARCP, annual review of competency progression

There were 5599 ARCPs (1464 CT/ST1–2 and 4135 HSTs) recorded in ISCP in 2019 and 5310 in 2020 (1307 CT/ST1–2 and 4003 HSTs). *Figures 2* and *3* represent the awarded ARCP outcomes by speciality in 2019 and 2020 respectively.

The median proportion of outcome 1 (trainees achieving competency at the expected rate) awarded across all grades and specialties in 2019 and 2020 was 63 (range 51–66) per cent and 47 (range 26–58) per cent respectively. The median proportion of outcomes 10.1 and 10.2 in 2020 were 15 per cent and 3 per cent respectively.

No significant difference was observed in the proportion of core trainees achieving an outcome 1 between 2019 and 2020. In 2020, 14 per cent (182 of 1307 trainees) received an outcome 10.1 and 2 per cent (22 of 1307) received an outcome 10.2.

A reduction in ARCP outcome 1 awarded to HSTs was observed. Twenty percent (795 trainees) of HSTs received an outcome 10.1 and 4 per cent (147 trainees) an outcome 10.2 in 2020. There was a reduction in outcomes 2, 3 and 5, whilst outcome 6 awards remained unchanged (*Table S2*). *Table 2* outlines the breakdown of COVID-19 related outcomes awarded to trainees at key progression points.

**Table 2:** 2020 COVID-related annual review of competency progression outcomes for trainee grades at key progression points

ARCP outcome	Trainee grades	
	–	**CT1/2**
**10.1**	–	182 of 1307 (14)
**10.2**	–	22 of 1307 (2)
	**ST7 (all specialties)**	**ST8 (all specialties)**
**10.1**	85 of 324 (26)	9 of 250 (4)
**10.2**	22 of 324 (7)	31 of 250 (12)

Values in parentheses are percentages. Urology and maxillofacial surgery are 7-year programmes and so 10.2 grades seen here are most likely certificate of completion of training (CCT) extensions in those specialties. ARCP, annual review of competency progression; CT, core training; ST, specialty training.

*Fig. S1a–j* outlines the impact of the COVID-19 pandemic on emergency and elective operative experience, regional operative variations and awarded ARCP outcomes by each surgical specialty.

## Discussion

This is the first quantitative study on the impact of the COVID-19 pandemic on surgical training on a national scale across all specialties. An overall negative impact has been observed with a reduction in over one third of logged cases in 2020 compared with those recorded in 2019. More than one in eight trainees in the final year have had their training extended through inability to achieve curricular requirements and more than a quarter entering their final year of training are behind their expected training trajectory. Although emergency cases involving trainees reduced in the early stages of the pandemic, they recovered to, or near to, baseline by the early summer of 2020. Most of the reduction in operative training was therefore seen in elective surgery. Throughout the duration of the pandemic to date, operative experience has not returned to baseline levels, with a further reduction as a result of the second wave of the pandemic. All regions of the UK and all surgical specialties have been severely affected, with the only differences being the degree of impact of COVID-19 in different parts of the country, largely reflecting pre-existing health resource inequalities. The current evidence base offers good insight into the qualitative impact on trainees during the pandemic. Cancellation of elective services has jeopardized competency attainment and progression in time-based training programmes in the UK, USA and Canada^19^ which are dependent upon exposure to and performance of indicative numbers. Additionally, while operative experience is only one aspect of surgical training, other areas of training have also been affected, including outpatient experience^20^, completion of courses and examinations and teaching^21^^,^^22^. The resultant impact on trainees’ mental health and wellbeing has also been significant^23^^,^^24^.

As the pandemic continues, it remains a challenge for trainees to regain the trajectory required to meet the outcomes of their curriculum, and many will require an extension to their training at their next assessment. Many who received a satisfactory ARCP outcome 1 this year may not meet the level of experience and competence suggested at the next progression point in their curriculum and are likely to be awarded outcome 10.1 or 10.2. Although it has been acknowledged that outcome 10.1 will not preclude progression from core surgical training to specialty training, the attainment gap that exists for those trainees will have to be addressed in the first year of specialty training.

Extension of training for a significant proportion of trainees has serious implications for workforce planning and service, as the supply of new consultants is delayed. This is particularly unwelcome at a time when early retirement of consultants is not slowing in the UK^25^. A substantial increase in the number of training places may be one solution but allowing potentially inexperienced trainees to progress, by lowering the standards required for completion of training, cannot be in the interests of patients, the service or trainees. The importance of surgical training to support existing surgical services and create a future workforce must be fully acknowledged by healthcare planners and is a vital lesson to be learned from this pandemic.

A framework to deal with these deficits in training has been suggested in a document developed jointly by the JCST, Association of Surgeons in Training, British Orthopaedic Trainees Association and The Confederation of Postgraduate Schools in Surgery^26^. Proposals include maintaining elective service delivery and training at COVID-light sites, minimizing redeployment of trainees in surgery, especially those at or approaching critical progression points in their training and increasing training opportunities especially when NHS patients are treated at independent hospitals. Multiple consultant reports and trainee self-assessment tools described in the surgical curricula from August 2021 should facilitate identification of bespoke training needs and the development of appropriate training action plans^27^. In other countries a personalized approach for additional training has been considered^9^. Curricular updates to reflect lost opportunities in addition to the utilization of virtual learning modalities in the form of simulation-based training may offer further solutions in surgical skill acquisition in a time of limited operative exposure^28^.

The limitations of this study include the period of data capture that included the changeover period of July/August when trainees historically took remaining leave allocations and moved to new posts with a seasonal fall in operative numbers. It is not clear if these leave and rotation patterns were the same for each of the study years. Urology and maxillofacial surgery have slightly shorter training programmes, so there is less certainty around the true proportion of trainees whose assessment at the end of their training delayed receipt of a certificate of completion of training (CCT).

The COVID-19 pandemic has had a major impact on training in surgery in the UK, which is consistent with the message globally. Urgent, coordinated action is required by all stakeholders to minimize the impact of this reduction in training in the future.

## Supplementary Material

zrab051_Supplementary_Data

## References

[1] Stevens S , Pritchard A. *Next steps on NHS Response to COVID-19*. https://www.england.nhs.uk/coronavirus/wp-content/uploads/sites/52/2020/03/urgent-next-steps-on-nhs-response-to-covid-19-letter-simon-stevens.pdf (accessed 11 December 2020)

[2] COVIDSurg Collaborative. Elective surgery cancellations due to the COVID-19 pandemic: global predictive modelling to inform surgical recovery plans. Br J Surg 2020;107:1440–1449 10.1002/bjs.11746 32395848 PMC7272903

[3] Aziz H , James T, Remulla D, Sher L, Genyk YS, Sullivan ME et al Effect of COVID-19 on surgical training across the United States: a national survey of general surgery residents. J Surg Educ 2020;30: 252–256. 10.1016/j.jsurg.2020.07.037 PMC7391955 32798154

[4] Osama M , Zaheer F, Saeed H, Anees K, Jawed Q, Syed SH et al Impact of COVID-19 on surgical residency programs in Pakistan; A residents' perspective. Do programs need formal restructuring to adjust with the ‘new normal’? A cross-sectional survey study. Int J Surg 2020;79:252–256 doi:10.1016/j.ijsu.2020. 06.004 32526265 10.1016/j.ijsu.2020.06.004 PMC7280820

[5] Lund J. Training during and after COVID-19. Bulletin 2020;102:10–13 doi:10.1308/rcsbull.TB2020.4

[6] Megaloikonomos PD , Thaler M, Igoumenou VG, Bonanzinga T, Ostojic M, Couto AF et al Impact of the COVID-19 pandemic on orthopaedic and trauma surgery training in Europe. Int Orthop 2020;44:1611–1619 doi:10.1007/s00264-020-04742-3 32696334 10.1007/s00264-020-04742-3 PMC7372204

[7] Adesunkanmi AO , Ubom AE, Olasehinde O, Wuraola FO, Ijarotimi OA, Okon NE et al Impact of the COVID-19 pandemic on surgical residency training: perspective from a low-middle income country. World J Surg 2021;45:10–17 doi:10.1007/s00268-020-05826-2 33118075 10.1007/s00268-020-05826-2 PMC7594960

[8] Hope C , Reilly JJ, Griffiths G, Lund J, Humes D. The impact of COVID-19 on surgical training: a systematic review. Tech Coloproctol 2021;25:505–520 doi:10.1007/s10151-020-02404-5 33507436 10.1007/s10151-020-02404-5 PMC7841379

[9] Zheng J , Hundeyin M, He K, Sachs T, Hess DT, Whang E et al General surgery chief residents' perspective on surgical education during the coronavirus disease 2019 (COVID-19) pandemic. Surgery 2020;168:222–225 doi:10.1016/j.surg.2020.06.003 32600881 10.1016/j.surg.2020.06.003 PMC7287487

[10] Pelargos PE , Chakraborty A, Zhao YD, Smith ZA, Dunn IF, Bauer AM. An evaluation of neurosurgical resident education and sentiment during the coronavirus disease 2019 pandemic: a North American Survey. World Neurosurg 2020;140:e381–e386 32512244 10.1016/j.wneu.2020.05.263 PMC7274118

[11] Marriott JC , Purdie H, Millen A, Beard JD. The lost opportunities for surgical training in the NHS. Bulletin 2011;93:202–206 doi:10.1308/147363511X57571

[12] Alderson D , Taylor J, Griffin SM, Mealy K. *Joint Policy Statement from the Royal Surgical Colleges*. https://www.rcsed.ac.uk/news-public-affairs/news/2020/march/joint-policy-statement-on-covid-19-from-the-royal-surgical-colleges (accessed 11 November 2020)

[13] Stevens S , Pritchard A. *Third Phase of NHS Response to COVID-19*. https://www.england.nhs.uk/coronavirus/wp-content/uploads/sites/52/2020/07/20200731-Phase-3-letter-final-1.pdf (accessed 19 December 2020)

[14] The Intercollegiate Surgical Curriculum. *Educating Surgeons of the Future*. 2013. https://www.iscp.ac.uk/static/public/syllabus/syllabus_gs_2016.pdf (accessed 11 November 2020)

[15] Conference of Postgraduate Medical Deans of the United Kingdom. *Implementing ARCP Outcomes 10.1 and 10.2 during COVID-19*. 2020. https://www.rcoa.ac.uk/sites/default/files/documents/2020-05/Implementing%20ARCP%20Outcomes%2010-1%20and%2010-2%20%20during%20COVID-19.PDF (accessed 11 November 2020)

[16] Conference of Postgraduate Medical Deans of the United Kingdom. *Derogation to Gold Guide 8th Edition: 4.91 in Response to COVID Pandemic and Impact on Trainee Progression Assessments (ARCP)*. https://www.copmed.org.uk/gold-guide-8th-edition/the-gold-guide-8th-edition (accessed 11 December 2020)

[17] Health Education England. *ARCP Outcomes*. https://heeoe.hee.nhs.uk/revalidation/assessment/arcp-outcomes (accessed 12 November 2020)

[18] Von Elm E , Altman DG, Egger M, Pocock SJ, Gotzsche PC, Vandenbroucke JP, STROBE Initiative. Strengthening the Reporting of Observational Studies in Epidemiology (STROBE) statement: guidelines for reporting observational studies. BMJ 2007;335:806–808 17947786 10.1136/bmj.39335.541782.AD PMC2034723

[19] James HK , Pattison GTR. Disruption to surgical training during Covid-19 in the United States, United Kingdom, Canada, and Australasia: a rapid review of impact and mitigation efforts. J Surg Educ 2021;78:308–314 doi:10.1016/j.jsurg.2020.06.020 32694085 10.1016/j.jsurg.2020.06.020 PMC7315967

[20] British Medical Association *. The Hidden Impact of COVID-19 on Patient Care in the NHS in England*. https://www.bma.org.uk/media/2841/the-hidden-impact-of-covid_web-pdf.pdf (accessed 11 December 2020)

[21] English W , Vulliamy P, Banerjee S, Arya S. Surgical training during the COVID-19 pandemic – the cloud with a silver lining? Br J Surg 2020 107:e343–e344 doi:10.1002/bjs.11801 32662537 10.1002/bjs.11801 PMC7404654

[22] Coleman JR , Abdelsattar JM, Glocker RJ; RAS-ACS COVID-19 Task Force. COVID-19 pandemic and the lived experience of surgical residents, fellows, and early-career surgeons in the American College of Surgeons. J Am Coll Surg 2021;232:119–135.e20 doi:10.1016/j.jamcollsurg.2020.09.026 33069850 10.1016/j.jamcollsurg.2020.09.026 PMC7561602

[23] Alhaj AK , Al-Saadi T, Mohammad F, Alabri S. Neurosurgery residents’ perspective on COVID-19: knowledge, readiness, and impact of this pandemic. World Neurosurg 2020;139:e848–e858 32426064 10.1016/j.wneu.2020.05.087 PMC7229445

[24] COVID-STAR Collaborative Study Group. COVID-19 impact on Surgical Training and Recovery Planning (COVID-STAR) – a cross-sectional observational study. Int J Surg 2021;88: 1–8. 10.1016/j.ijsu.2021.105903 PMC7912362 33652133

[25] British Medical Association. *Consultant Workforce Shortages and Solutions – Now and in the Future*. https://www.bma.org.uk/media/3430/bma-consultants-retention-paper.pdf (accessed 11 December 2020)

[26] JCST, ASiT, BOTA and CoPSS. *Maximising Training Opportunities*. https://www.jcst.org/jcst-news/2020/11/10/news-item/ (accessed on 18 November 2020)

[27] Joint Committee on Surgical Training (JCST). *The New Surgical Curriculum for August 2021*. https://www.iscp.ac.uk/iscp/curriculum-2021/ (accessed 11 December 2020)

[28] Stambough JB , Curtin BM, Gililland JM, Guild GN, Kain MS, Karas V et al The past, present, and future of orthopedic education: lessons learned from the COVID-19 pandemic. J Arthroplasty 2020;35:S60–S64 doi:10.1016/j.arth.2020.04.032 10.1016/j.arth.2020.04.032 PMC7166110 32345564

