# Supplementary material for: The quantitative impact of COVID-19 on surgical training in the United Kingdom
Source: BJS Open. 2021 Jun 25;5(3):zrab051. doi: 10.1093/bjsopen/zrab051 (PMC8226285; doi:10.1093/bjsopen/zrab051)
Supplement: zrab051_Supplementary_Data [file zrab051_supplementary_data.zip › Supplementary_table_1_Clements (1).docx]

| **ARCP Outcome** | **Definition** | |
| --- | --- | --- |
| Outcome 1 | Satisfactory Progress – Achieving progress and the development of competencies at the expected rate. | |
| Outcome 2 | Development of specific competencies required – additional training time not required | |
| Outcome 3 | Inadequate progress - additional training time required (extension of anticipated core training programme end date or anticipated CCT/CESR(CP)/CEGPR(CP) date) | |
| Outcome 4 | Released from training programme – with or without specified competencies | |
| Outcome 5 | Incomplete evidence presented - additional training time may be required.    If you receive an outcome 5, you will be asked to provide additional information within a specific, short-term timeframe. After presentation of the extra evidence, the ARCP panel will update your ARCP outcome to either an outcome 1, 2, 3, 4, 6 or 8 depending on the additional evidence you have provided. | |
| Outcome 6 | Gained all required competencies - will be recommended as having completed the training programme (core or specialty) and if in a run-through training programme or higher training programme, will be recommended for award of CCT or CESR(CP)/CEGPR(CP) | |
| Outcome 7  FTSTA or LAT Trainees | 7.1 | Satisfactory progress for completion of the post |
|  | 7.2 | Development of specific competencies required – additional time not required |
|  | 7.3 | Inadequate progress by the trainee (progress not sufficient for period of training to be formally recognised towards CCT/CESR(CP)/CEGPR(CP) or full CESR/CEGPR) |
|  | 7.4 | Incomplete evidence presented |
| Outcome 8 | Out of programme for research, approved clinical training or a career break (OOPR/OOPT/OOPC | |
| Not assessed | There are circumstances when an ARCP panel is not able to recommend an outcome. For example, if a trainee is absent due to statutory leave. In these cases, the ARCP panel will record the reason why no ARCP outcome could be recommended | |

*Supplementary Table 1 | Standard ARCP outcomes ^14^*

*^14^* Health Education England. ARCP outcomes. <https://heeoe.hee.nhs.uk/revalidation/assessment/arcp-outcomes> [Accessed 12th November 2020]
