# Supplementary material for: The quantitative impact of COVID-19 on surgical training in the United Kingdom
Source: BJS Open. 2021 Jun 25;5(3):zrab051. doi: 10.1093/bjsopen/zrab051 (PMC8226285; doi:10.1093/bjsopen/zrab051)
Supplement: zrab051_Supplementary_Data [file zrab051_supplementary_data.zip › Supplementary_table_2_Clements (1).docx]

|  | **1** | **10.1** | **10.2** | **2** | **3** | **4** | **5** | **6** | **7** | **8** | **No outcome** | **Nil recorded** | **Total** |
| --- | --- | --- | --- | --- | --- | --- | --- | --- | --- | --- | --- | --- | --- |
| **Core Training (CT)** | 493 | 0 | 0 | 118 | 84 | 17 | 249 | 459 | 0 | 8 | 29 | 7 | 1464 |
| **Cardiothoracic Surgery** | 76 | 0 | 0 | 6 | 9 | 0 | 10 | 14 | 1 | 8 | 1 | 17 | 142 |
| **General Surgery** | 671 | 0 | 0 | 73 | 64 | 1 | 117 | 75 | 19 | 141 | 30 | 76 | 1267 |
| **Neurosurgery** | 169 | 0 | 0 | 13 | 9 | 0 | 19 | 17 | 1 | 24 | 3 | 7 | 262 |
| **OMFS** | 79 | 0 | 0 | 7 | 6 | 0 | 6 | 10 | 0 | 2 | 3 | 6 | 119 |
| **Otolaryngology** | 288 | 0 | 0 | 12 | 9 | 0 | 38 | 38 | 1 | 21 | 12 | 34 | 453 |
| **Paediatric Surgery** | 60 | 0 | 0 | 4 | 3 | 0 | 8 | 11 | 0 | 6 | 2 | 3 | 97 |
| **Plastic Surgery** | 127 | 0 | 0 | 7 | 7 | 0 | 13 | 12 | 2 | 12 | 5 | 15 | 200 |
| **Trauma & Orthopaedics** | 726 | 0 | 0 | 78 | 27 | 3 | 67 | 109 | 8 | 49 | 9 | 39 | 1115 |
| **Urology** | 153 | 0 | 0 | 31 | 8 | 2 | 18 | 37 | 2 | 15 | 14 | 20 | 300 |
| **Vascular Surgery** | 110 | 0 | 0 | 6 | 4 | 1 | 20 | 12 | 0 | 25 | 1 | 1 | 180 |
| **All (excluding Core)** | 2459 | 0 | 0 | 237 | 146 | 7 | 316 | 335 | 34 | 303 | 80 | 218 | 4135 |

*Supplementary Material 2a | All Awarded ARCP in 2019*

|  | **1** | **10.1** | **10.2** | **2** | **3** | **4** | **5** | **6** | **7** | **8** | **No outcome** | **Nil recorded** | **Total** |
| --- | --- | --- | --- | --- | --- | --- | --- | --- | --- | --- | --- | --- | --- |
| **Core training (CT)** | 436 | 182 | 22 | 70 | 43 | 14 | 114 | 389 | 0 | 14 | 15 | 8 | 1307 |
| **Cardiothoracic Surgery** | 36 | 31 | 20 | 1 | 3 | 0 | 7 | 5 | 1 | 14 | 1 | 19 | 138 |
| **General Surgery** | 557 | 177 | 40 | 43 | 36 | 0 | 78 | 75 | 8 | 100 | 45 | 56 | 1215 |
| **Neurosurgery** | 145 | 17 | 8 | 7 | 6 | 0 | 7 | 14 | 1 | 27 | 2 | 14 | 248 |
| **OMFS** | 73 | 16 | 6 | 0 | 5 | 0 | 10 | 9 | 0 | 5 | 4 | 4 | 132 |
| **Otolaryngology** | 197 | 84 | 11 | 7 | 1 | 1 | 8 | 26 | 3 | 17 | 14 | 17 | 386 |
| **Paediatric Surgery** | 31 | 9 | 8 | 5 | 3 | 0 | 9 | 1 | 0 | 9 | 4 | 9 | 88 |
| **Plastic Surgery** | 105 | 24 | 4 | 2 | 5 | 0 | 13 | 21 | 1 | 7 | 7 | 13 | 202 |
| **Trauma and orthopaedics** | 407 | 358 | 42 | 42 | 15 | 3 | 48 | 120 | 10 | 36 | 10 | 53 | 1144 |
| **Urology** | 134 | 43 | 6 | 8 | 3 | 1 | 9 | 30 | 4 | 15 | 10 | 13 | 276 |
| **Vascular Surgery** | 76 | 36 | 2 | 3 | 2 | 0 | 5 | 11 | 2 | 24 | 1 | 12 | 174 |
| **All (excluding Core)** | 1761 | 795 | 147 | 118 | 79 | 5 | 194 | 312 | 30 | 254 | 98 | 210 | 4003 |

*Supplementary Material 2b | All Awarded ARCP in 2020*
