# Supplementary figures and images for: The quantitative impact of COVID-19 on surgical training in the United Kingdom
Source: BJS Open. 2021 Jun 25;5(3):zrab051. doi: 10.1093/bjsopen/zrab051 (PMC8226285; doi:10.1093/bjsopen/zrab051)

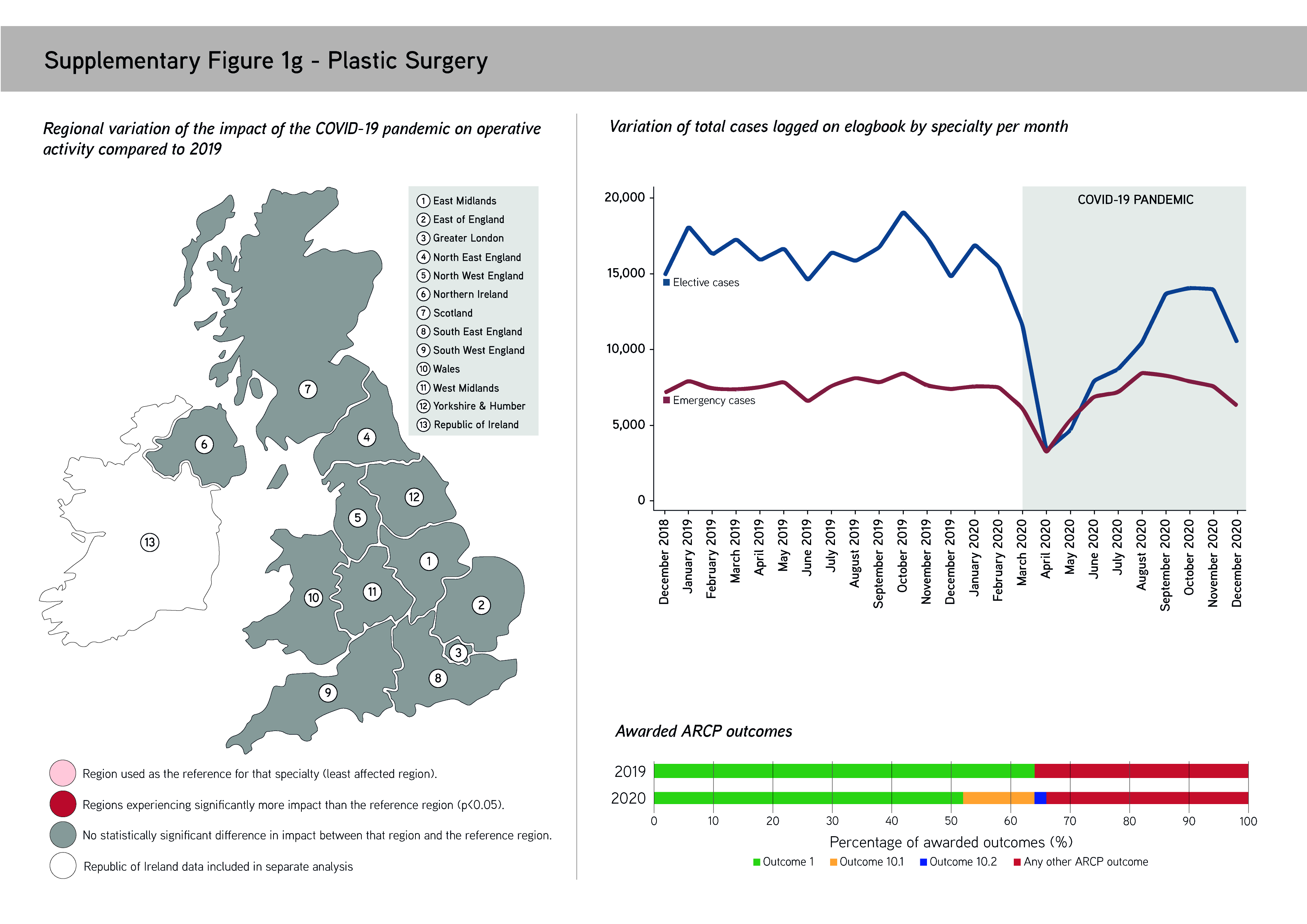

Supplement: zrab051_Supplementary_Data [file zrab051_supplementary_data.zip › Supplementary Figure 1g - Plastic Surgery.tiff]

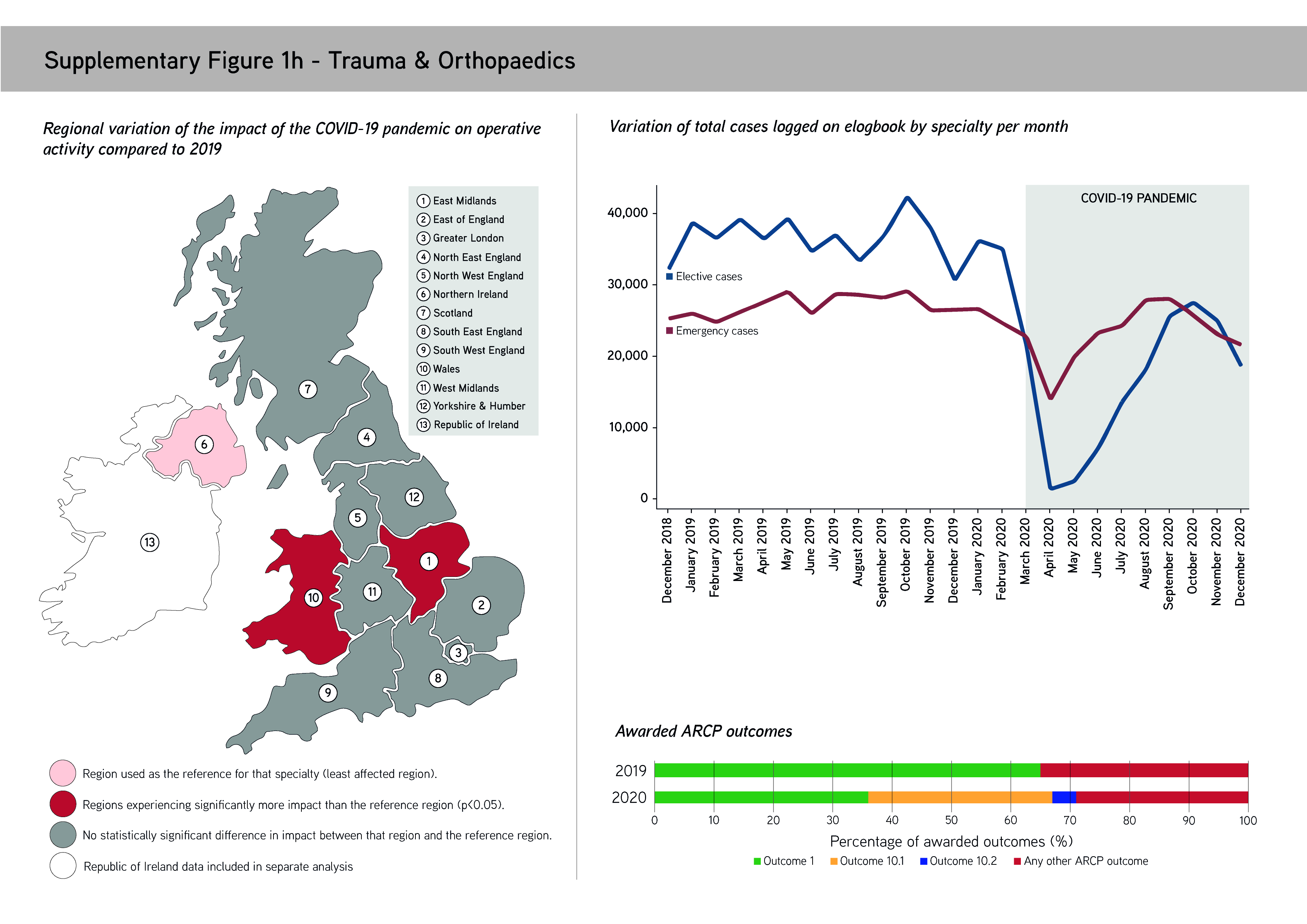

Supplement: zrab051_Supplementary_Data [file zrab051_supplementary_data.zip › Supplementary Figure 1h - Trauma _ Orthopaedics.tiff]

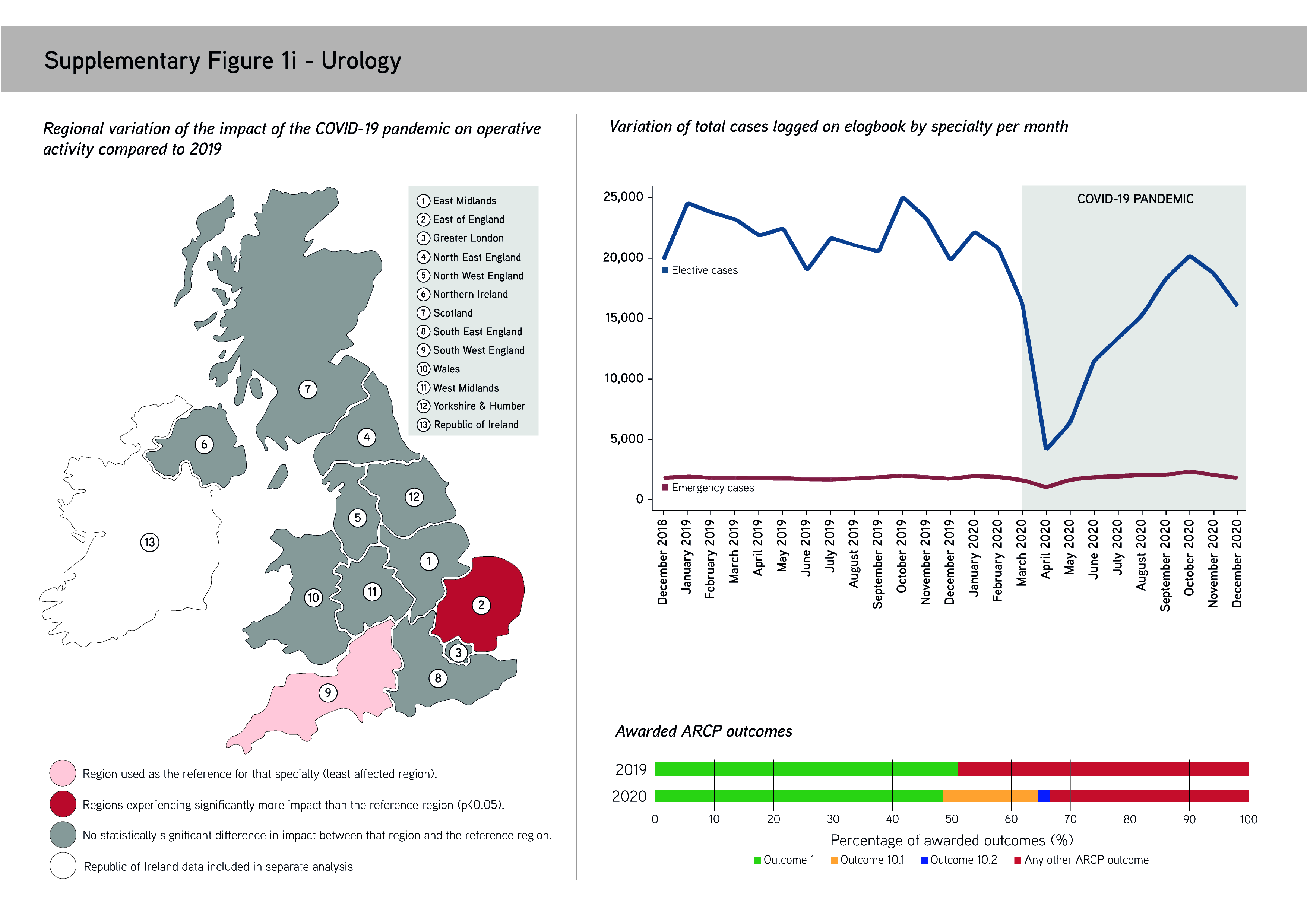

Supplement: zrab051_Supplementary_Data [file zrab051_supplementary_data.zip › Supplementary Figure 1i - Urology.tiff]

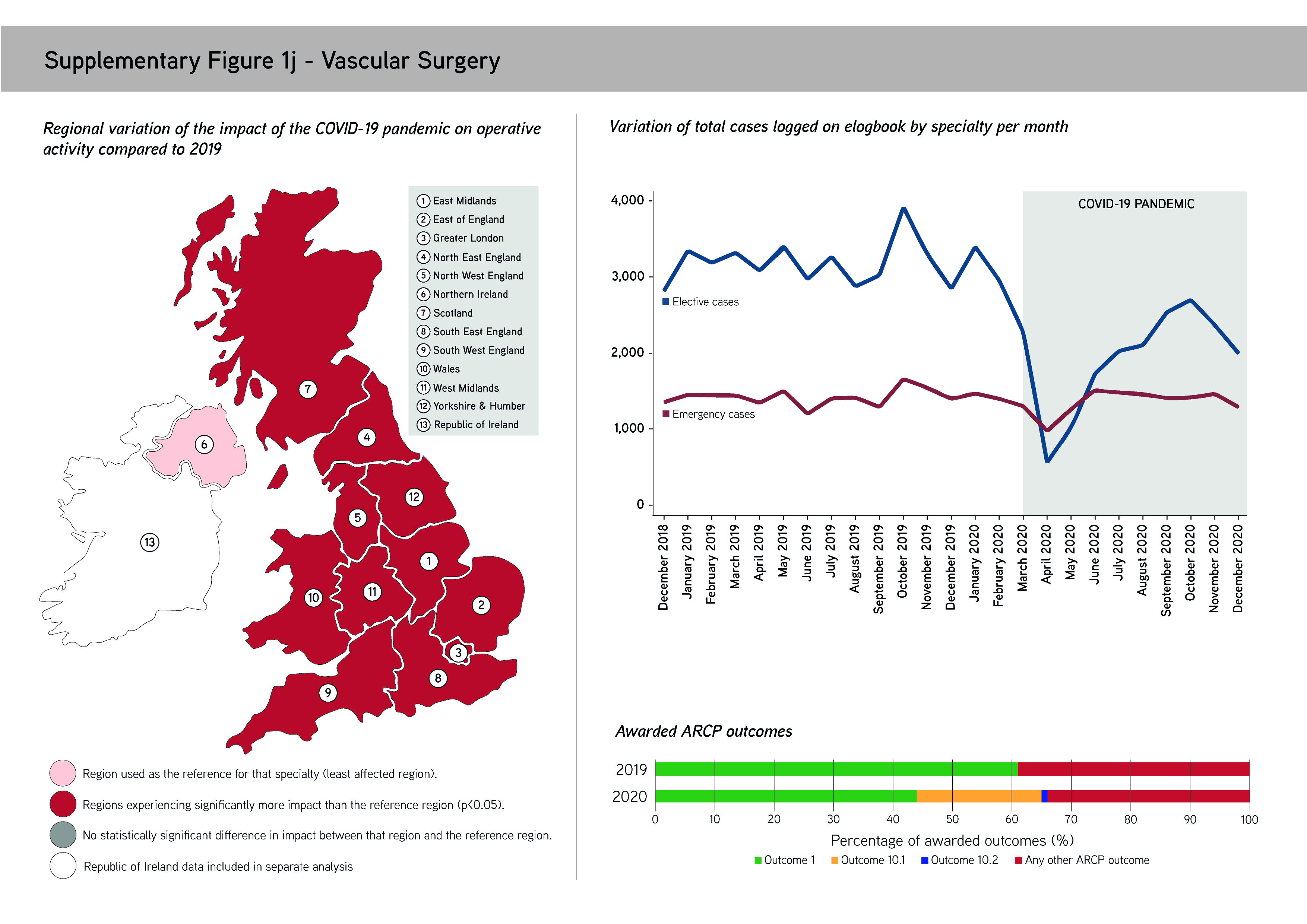

Supplement: zrab051_Supplementary_Data [file zrab051_supplementary_data.zip › Supplementary Figure 1j - Vascular Surgery.tiff]

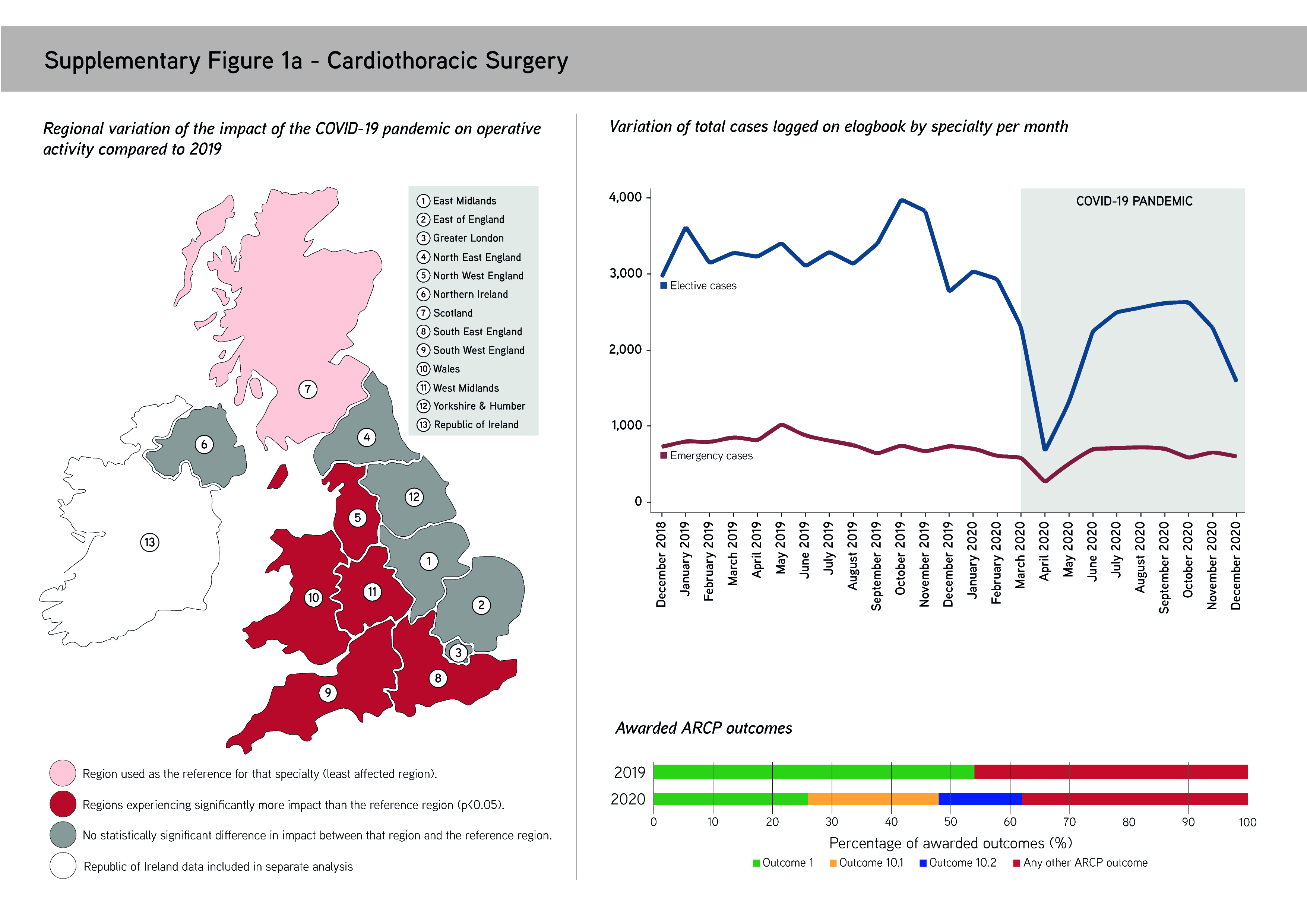

Supplement: zrab051_Supplementary_Data [file zrab051_supplementary_data.zip › Supplementary Figure 1a - Cardiothoracic surgery.tiff]

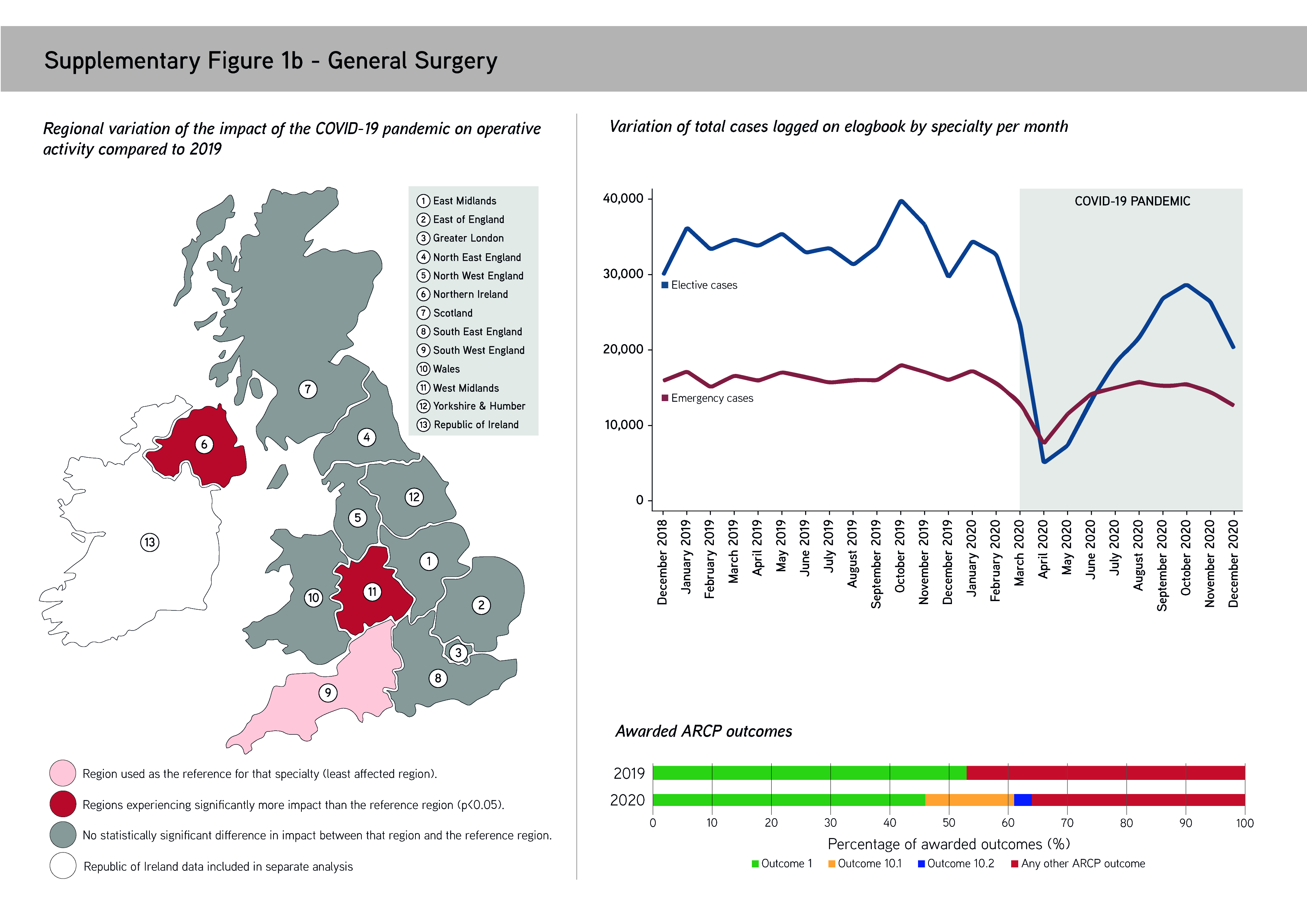

Supplement: zrab051_Supplementary_Data [file zrab051_supplementary_data.zip › Supplementary Figure 1b - General Surgery.tiff]

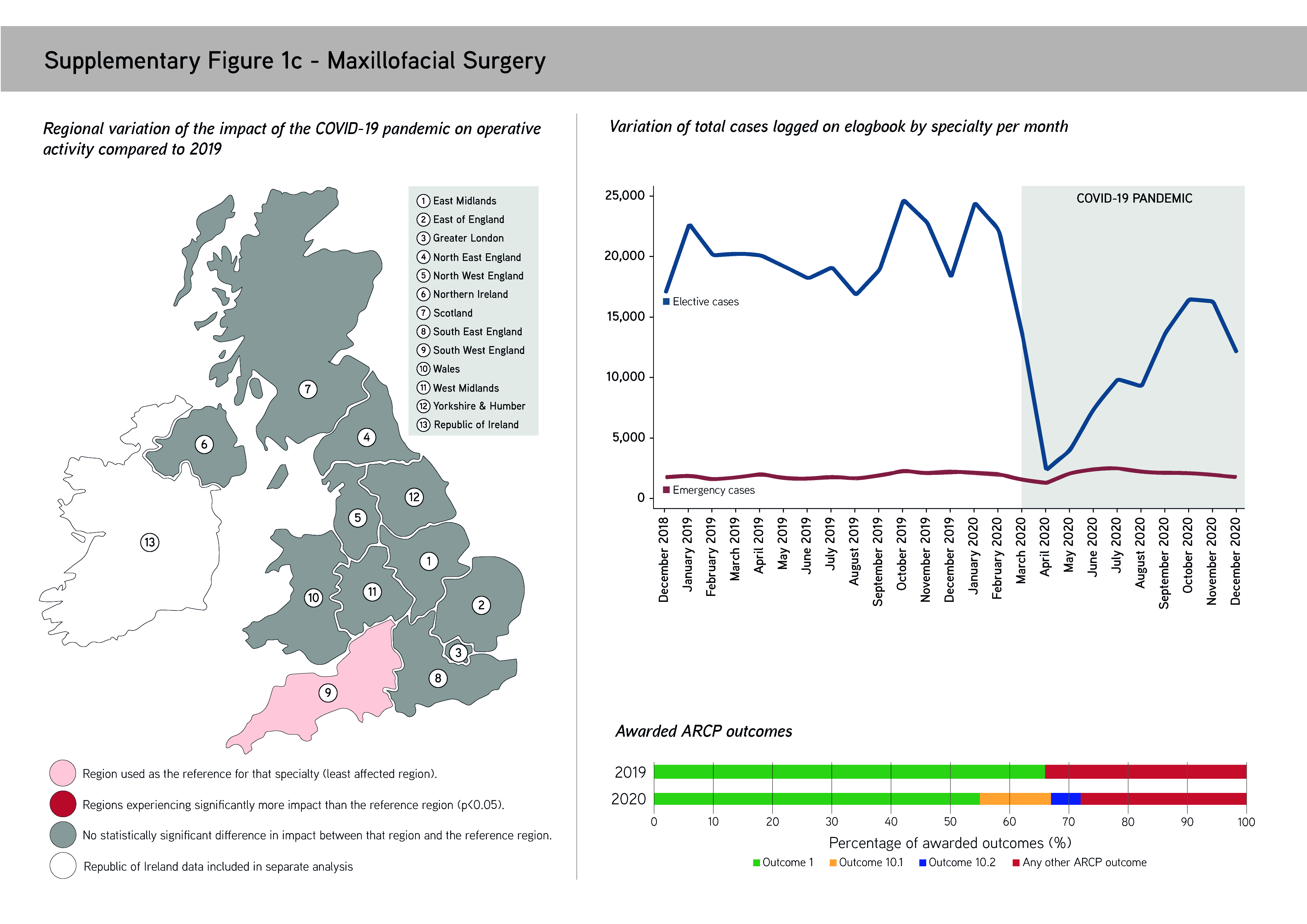

Supplement: zrab051_Supplementary_Data [file zrab051_supplementary_data.zip › Supplementary Figure 1c - Maxillofacial Surgery.tiff]

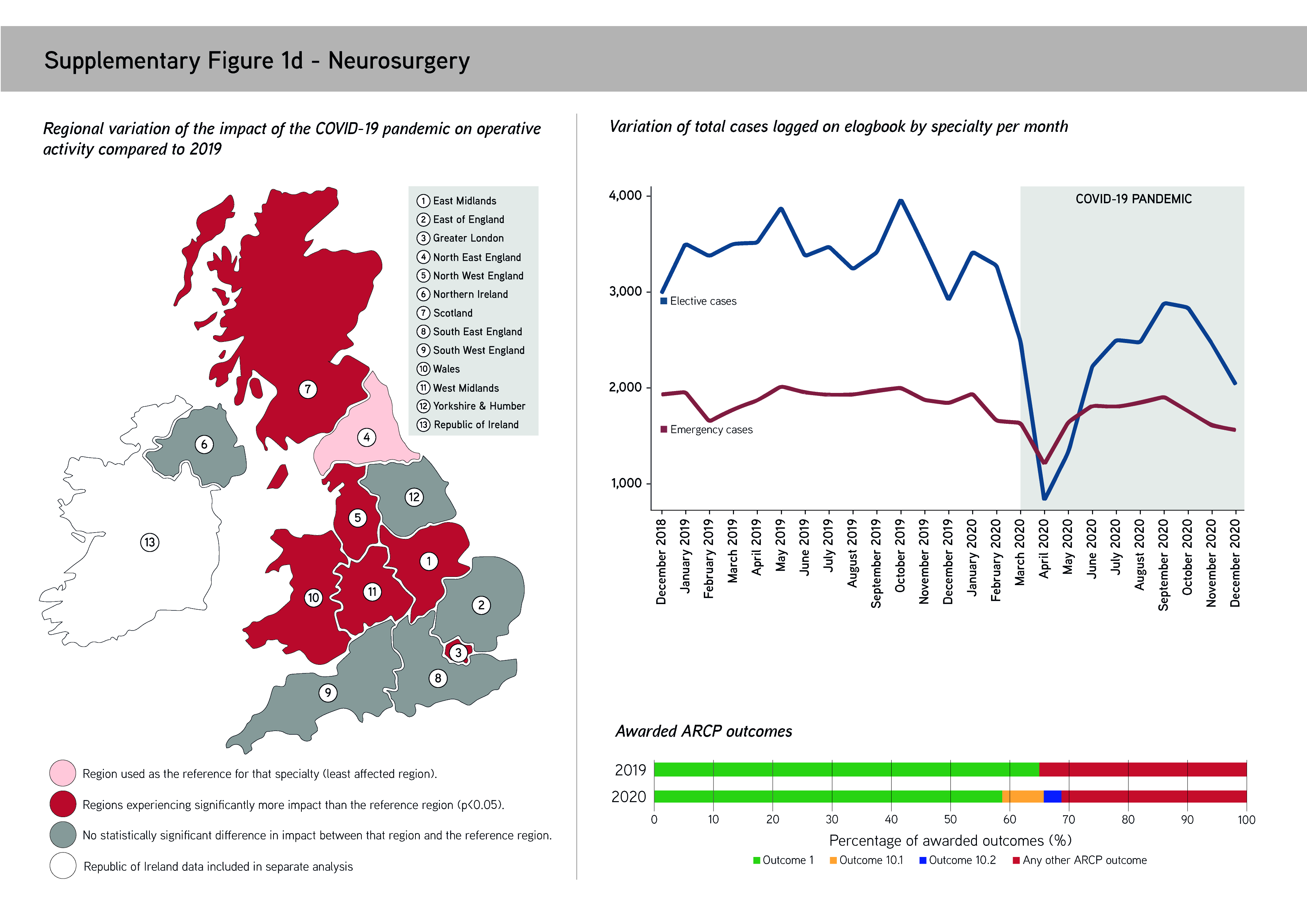

Supplement: zrab051_Supplementary_Data [file zrab051_supplementary_data.zip › Supplementary Figure 1d - Neurosurgery.tiff]

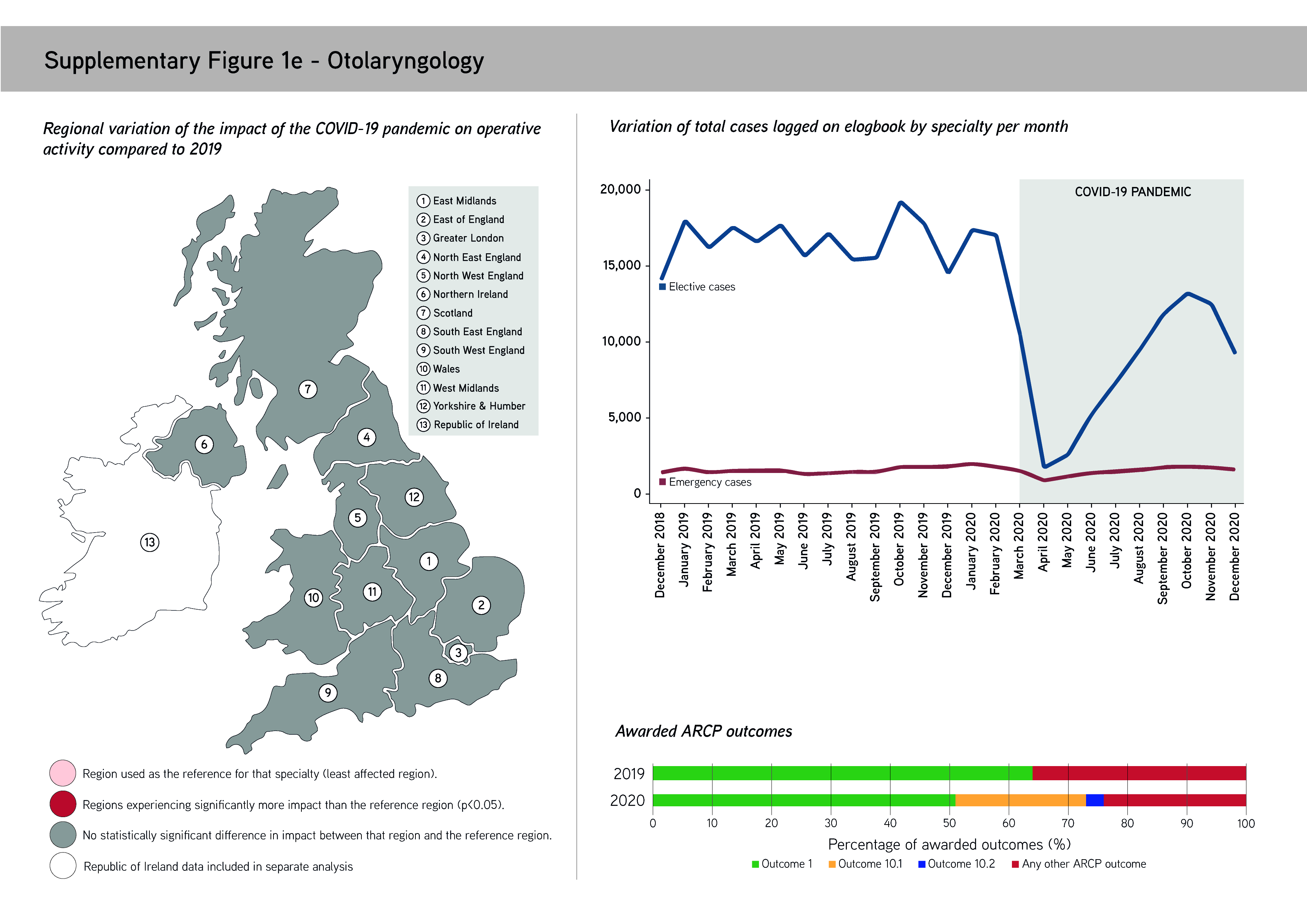

Supplement: zrab051_Supplementary_Data [file zrab051_supplementary_data.zip › Supplementary Figure 1e - Otolaryngology.tiff]

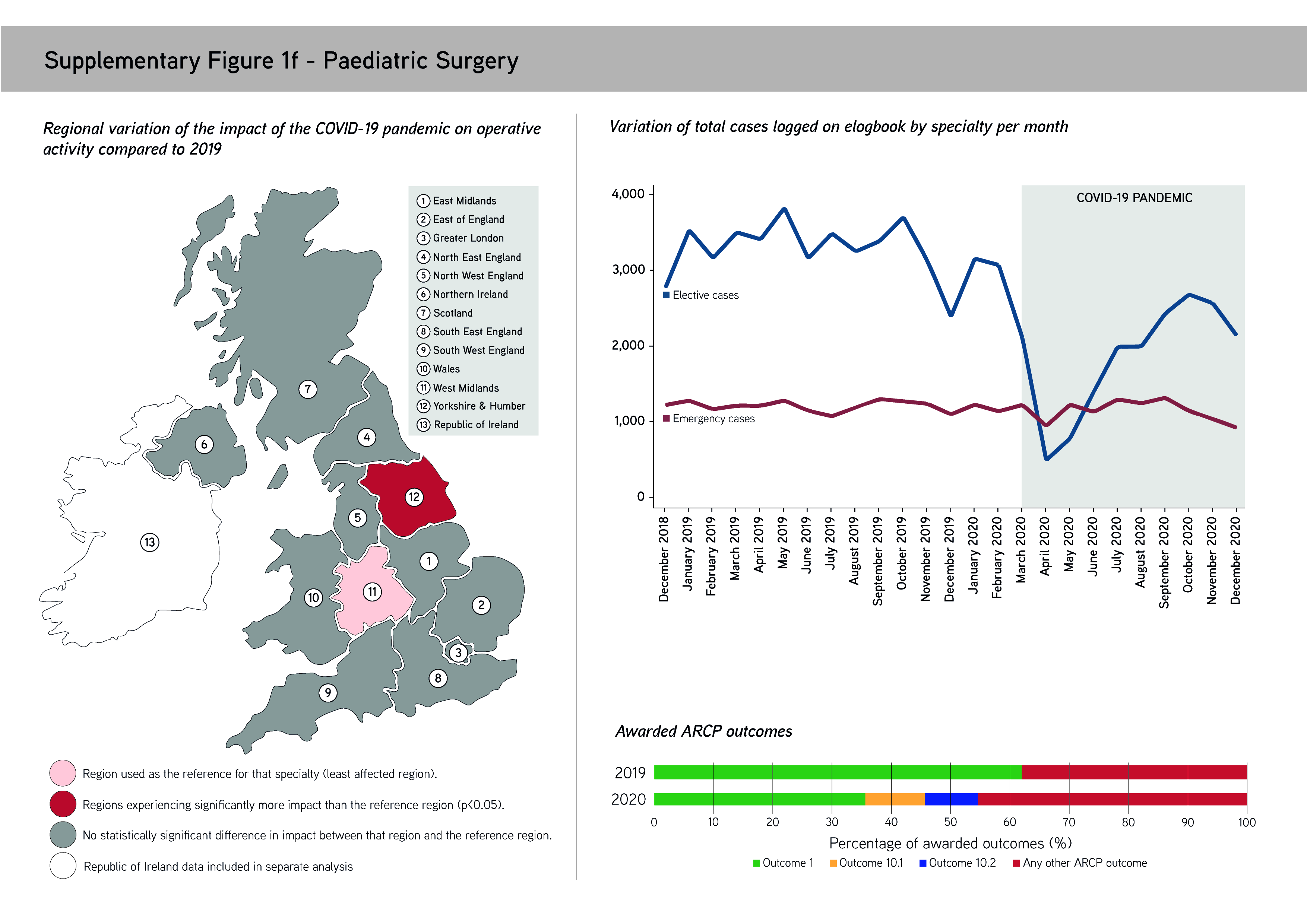

Supplement: zrab051_Supplementary_Data [file zrab051_supplementary_data.zip › Supplementary Figure 1f - Paediatric Surgery.tiff]
